# Lymphocyte migration regulation related proteins in urine exosomes may serve as a potential biomarker for lung cancer diagnosis

**DOI:** 10.1186/s12885-023-11567-x

**Published:** 2023-11-18

**Authors:** Shuai Jin, Tianci Liu, Weiwei Wang, Tao Li, Zhefeng Liu, Man Zhang

**Affiliations:** 1 Senior Department of Oncology, the Fifth Medical Center of PLA General Hospital, Beijing, 100071 China; 2 Beijing Key Laboratory of Urinary Cellular Molecular Diagnostics, Beijing, 100038 China; 3 Clinical Laboratory Medicine, Beijing Shijitan Hospital, Capital Medical University, Beijing, 100038 China; 4 Department of Pulmonary and Critical Care Medicine, Beijing Shijitan Hospital, Capital Medical University, Beijing, 100038 China; 5 Institute of Regenerative Medicine and Laboratory Technology Innovation, Qingdao University, Qingdao, 266071 China

**Keywords:** Lung cancer, Urine exosomes, Lymphocyte migration, Biomarkers

## Abstract

**Background:**

The migration of lymphocytes shares many similarities in mode and mechanism with the metastasis of lung cancer tumor cells. But changes in the expression of lymphocyte migration regulation related proteins in urine exosomes remain unclear. This study is to investigate the expression changes of lymphocyte migration regulation related proteins in urine exosomes of lung cancer patients, and further verify their correlation with the development and progression of lung cancer.

**Methods:**

Urine exosomes were collected from lung cancer patients and healthy people aged 15–79 years. Mass spectrometry was used to screen and explore the expression changes of lymphocyte migration regulation related proteins in healthy people of different ages. Enzyme-linked immunosorbent assay and western blotting were used to detect the expression changes of lymphocyte migration regulation related proteins in lung cancer patients.

**Results:**

Analyzing the data of urine exosome proteomics, a total of 12 lymphocyte related proteins were identified, 5 of which were lymphocyte migration regulation related proteins. Among these proteins, WASL and STK10 proteins showed a gradual decrease in expression with age, and WNK1 protein showed a gradual increase. Lung cancer patients had reduced expression of WASL and increased expression of STK10 and WNK1 proteins in urine exosomes compared to normal people. Urine exosome WASL, STK10, and WNK1 were diagnosed with lung cancer, with a combined AUC of 0.760.

**Conclusions:**

Lymphocyte migration regulation related proteins were differentially expressed in the urine exosome of lung cancer patients, and WASL, STK10 and WNK1 may serve as potential biomarkers for lung cancer diagnosis.

**Supplementary Information:**

The online version contains supplementary material available at 10.1186/s12885-023-11567-x.

## Introduction

Lung cancer is the most common form of cancer worldwide and originates in the epithelial cells of the respiratory tract [1]. In 2018, approximately 2.1 million patients were diagnosed with lung cancer, accounting for 12% of all cancers [2]. Lung cancer can be divided into two main categories, namely small cell lung cancer (SCLC) and non-small cell lung cancer (NSCLC) [3, 4]. The latter includes adenocarcinoma, squamous cell carcinoma, and large cell carcinoma, accounting for approximately 85% of lung cancers [5]. At present, the diagnosis and prognosis of lung cancer remain poor, and the survival rate of lung cancer has only slightly improved over the past few decades [6]. For example, data show that the average five-year survival rate for lung cancer patients in the United States is 19% [7]. Many patients are not diagnosed until the middle and late stages of the disease, so there is an urgent need to find new tumor markers and more sensitive indicators for assessing patient prognosis to find new targets and directions for lung cancer diagnosis and treatment.

Exosomes are tiny vesicles that can be secreted by most cells in the body and range in diameter from 30 to 140 nm. Its content is abundant, including proteins, lipids, DNA, and RNA [8]. Exosomes can mediate intercellular communication and signal transduction, playing important roles in growth and development [9], immune regulation [10] and other processes. With the exploration of the therapeutic potential of exosomes, exosomes have now completed phase I and II clinical trials in some patients with advanced stages of cancer, with extensive application prospects [11, 12]. Urine collection is simple and non-invasive. Urine exosomes can reflect the pathological state of the body, and as a non-invasive alternative to diagnostic testing, urine exosome is expected to be a non-invasive and convenient biomarker for early detection of lung cancer [13].

Changes in human immune function play an extremely important role during the clinical diagnosis and treatment of cancer [14]. It has been shown that the effective response of the immune system depends on the regulation of lymphocyte migration and homing in many ways. Mature T lymphocytes that have not yet been exposed to the antigen rely on homing receptors on their cell surface to colonize the thymus-dependent zones of the peripheral lymphoid organ. Proliferate and differentiate into effector T cells and memory T cells after being stimulated by the corresponding antigens. Thus, lymphocyte plays an important role in the human immune system. Lymphocyte migration is characterized by extensive and multisite migration, which makes it important in the pathogenesis of many diseases [15, 16]. Exploring the migration of lymphocytes in lung cancer patients has important implications for the diagnosis and treatment of lung cancer.

In this study, based on exosomes, we investigated the expression changes of lymphocyte migration regulation related proteins in healthy people at different ages according to the proteomics of urine exosomes from healthy people. Afterwards, the expression changes of proteins related to lymphocyte migration regulation in the urine exosome of lung cancer patients were explored to further validate their relevance to the pathogenesis of lung cancer, thereby laying the foundation for research on the diagnosis of lung cancer and the selection of therapeutic targets.

## Methods

### Patients

All study subjects gave informed consent before being included. All procedures were carried out in accordance with the ethical standards of the Helsinki Declaration and were approved by the ethics committee. In this study, lung cancer patients and physical examination population from February 2022 to September 2022 were collected as subjects. Normal controls were selected from the population with normal physical examinations, and diseases such as hypertension, diabetes and cancer were excluded. The clinical data of the subjects were collected retrospectively and all subjects were free of hematuria, proteinuria and ketosis.

The first cohort consisted of healthy people aged 15–79 years and was divided into 4 groups (15–30,31–44,45–59,60–79) with 30 people in each group. This group was used for mass spectrometry to screen and explore changes in the expression of proteins related to lymphocyte migration regulation at different ages. See Supplementary Table 1 for specific clinical information. Another cohort consisted of 44 lung cancer patients and 30 age-sex matched normal controls to explore and verify the expression changes of lymphocyte migration regulation related proteins in lung cancer patients.

### Exosome extraction

Collected 30 mL of clean morning urine from the subjects, and sent it to the laboratory within 2 h. After centrifugation at 1500 g for 10 min and 10,000 g for 30 min, dead cells and debris were removed. Then, the supernatant was filtered through a 0.22 μm filter to remove bacteria (Millipore, SLGVR33RB). Urine was concentrated by ultrafiltration tubes (Millipore, UFC910024) and exosomes were later collected using size exclusion SEC (qEV10 / 35 nm, IZON, Shanghai, China) [17, 18]. Finally, urine exosomes were resuspended in approximately 1 mL PBS and stored directly at -80 °C until use.

### Identification of urine exosomes

According to the guidelines of the International Society for Extracellular Vesicles (ISEV) for the characterization of exosomes, the morphology of exosomes was examined by transmission electron microscopy (TEM), and the purity and identity were analyzed by western blotting according to the expression of exosome markers.

### TEM

Five μL urine exosome sample was dropped on the sample-carrying copper grid, and after standing at room temperature for 5 min, the liquid was blotted dry from the side of the filter screen with filter paper. A drop of 2% uranyl acetate was then dropped on the sample, incubated for 1 min at room temperature, and the surface liquid was blotted with filter paper. After drying at room temperature, the morphology of urine exosomes was observed under a microscope (Tecnai G2 Spirit BioTwin, FEI).

### Western blotting

To further explore the expression of proteins in the urine exosome, we performed western blotting experiments. Lysis of urine exosome samples for at least 30 min under the RIPA lysis (strong) buffer containing PMSF. The concentration of protein samples was determined by the bicinchoninic acid (BCA) method. A total of 8 μg urine proteins were loaded on 8∼ 15% sodium dodecyl sulfate–polyacrylamide gel. Transferred to PVDF membrane by Trans-Blot Turbo Transfer System (Bio-Rad, California, USA), and shaken for 2 h at room temperature after blocking with 5% skim milk in TBST. Anti-CD9 (Abcam, ab236630, Non reducing conditions), Anti-CD63 (Proteintech, Cat No. 25682–1-AP, Non reducing conditions), Anti- Calnexin (Proteintech, Cat No. 10427–2-IG), Anti-WASL (Proteintech, Cat No. 14306–1-AP), Anti-STK10 (Affinity, Cat No. #DF4783), and Anti-WNK1 (Proteintech, Cat No. 28357–1-AP) were diluted at a ratio of 1:1000 ∼ 3000, respectively, and followed by incubation in a refrigerator at 4 °C. Washed three times with TBST for 15 min each time, added a 1:3000 diluted secondary antibody (HRP conjugated anti-Rabbit IgG, Lot:158,560), and incubated at room temperature for 1.5 h. After that, they were washed three times with TBST and detected by enhanced chemiluminescence (ECL, Bio-Rad, cat. #170–5061). The protein gel electrophoresis image was collected and analyzed by automatic chemiluminescence image analyzer (GenoSens 2000, QinXiang, Shanghai, China). Grayscale analysis of the western blotting bands was analyzed using the ImageJ software.

### Mass spectrometry analysis of urine exosomes

This study prepared mobile phase solution A (100% MS water, 0.1% formic acid) and solution B (100% acetonitrile, 0.1% formic acid). Peptides were separated in an analytical column using a linear gradient elution method. Experiments were performed on a QExactive HF-X mass spectrometer (Thermo Fisher), and mass spectra were acquired in data independent acquisition (DIA) mode with a full scan range of m/z 350–1500 and resolution of 120,000 (m/z 200). The automatic gain control target value was 2 × 10^5^, NanosprayFlex™ (ESI) ion source, and the ion spray voltage was set to 2.4 kV. Finally, the raw data of mass spectrometry detection were generated. Mass spectrometry results were queried in the SwissProt human database of UniProt (www.uniprot.org) using the proteome discovery software suite (Thermo Fisher Scientific v2.1). At the protein level, each protein contained at least one unique peptide using false discovery rate (FDR) of 1% as a filter.

### ELISA

Urine exosomes from lung cancer patients and normal controls were used as validation specimens. Urine exosome samples were lysed with RIPA strong lysis buffer, and protein concentrations were measured by the BCA method. The total amount of immobilized protein was 10 μg, and the sample volume was adjusted to 100 μL with sample buffer. ELISA kits from YuanJu Biotechnology Center (Shanghai, China; WASL: Cat No.YJ695541; STK10: Cat No.YJ396254; WNK1: Cat No.YJ032216) were used. According to the instructions, each sample was measured 3 times, and the unknown sample concentration was calculated according to the standard curve.

### Statistics

All experimental data were analyzed using GraphPad Prism 8.0 (GraphPad, La Jolla, CA, USA) statistical software. Differences between groups were compared using Student’s t-test. The diagnostic performance of proteins was evaluated using receiver operating characteristic curve (ROC) analysis, and binary logistic regression analysis was employed for joint diagnosis. Online resource gene ontology was utilized to investigate the signaling pathways of candidate biomarkers (http://geneontology.org). *P* < 0.05 was considered statistically significant.

## Results

### Clinical characteristics

**Fig. 1:**
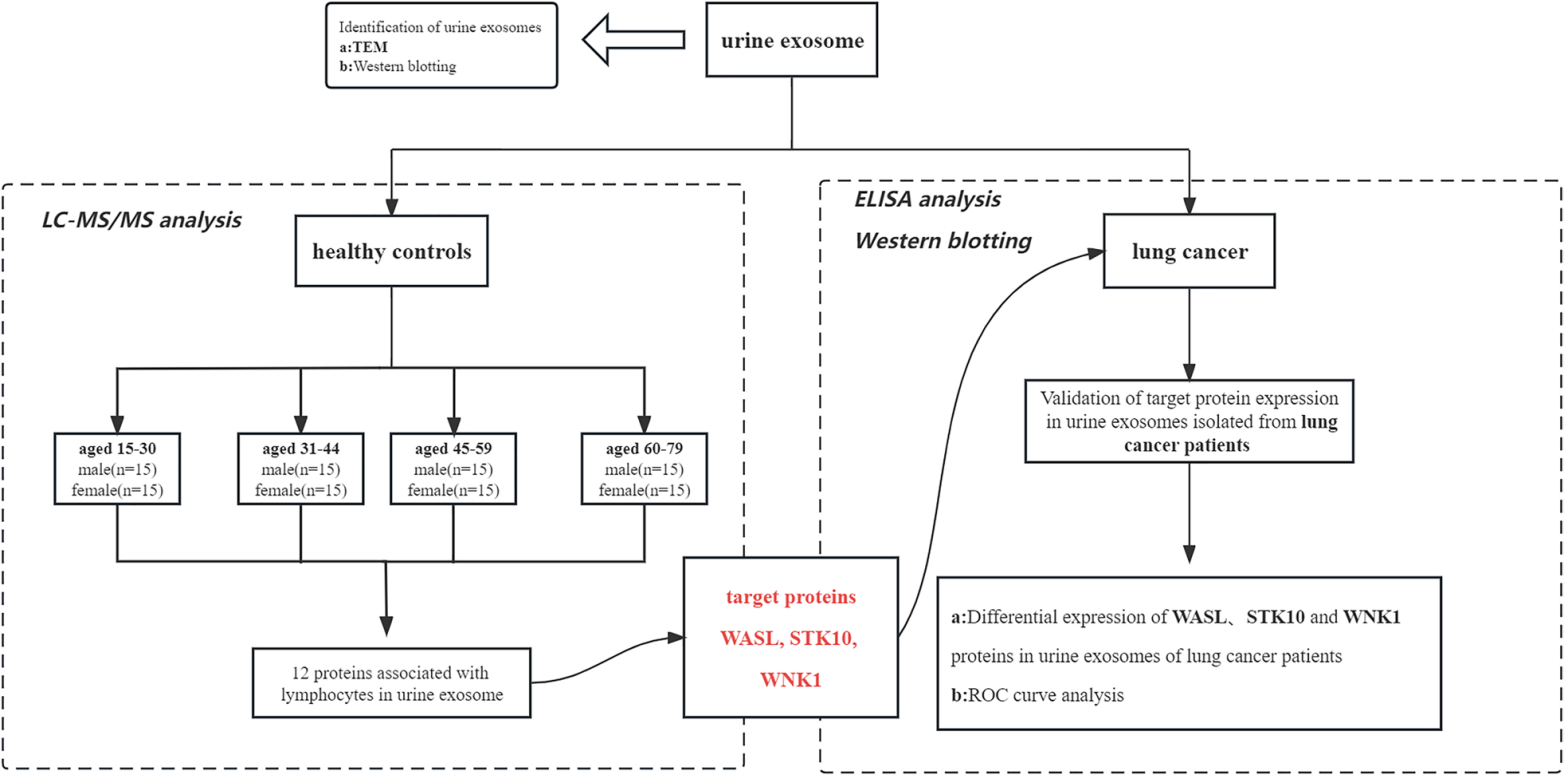
The detailed workflow of this study

The clinical data of 44 lung cancer patients and 30 age-sex matched normal controls are shown in Table 1. There were no statistical differences between lung cancer patients and normal controls in aspartate aminotransferase (AST), alanine aminotransferase (ALT), albumin (ALB), fasting blood glucose (FBG), and serum creatinine (Cr) (*P* > 0.05). The detailed workflow of this study is shown in Fig. 1.

**Table 1:** Demographic and clinical characteristics of lung cancer (LC) and normal control (NC)

Characteristics	LC (*n* = 44)	NC (*n* = 30)	*P* value
Age, years	59.98 ± 8.58	56.77 ± 4.82	ns
Gender; Male/female	31/13	20/10	NA
WBC × 10^9^/L	6.91 ± 5.34	6.33 ± 1.27	ns
RBC × 10^12^/L	3.80 ± 0.64	5.13 ± 0.36	^***^
Hb, g/L	113.25 ± 18.52	160.9 ± 8.32	^***^
NE, %	62.37 ± 14.15	58.36 ± 8.42	ns
LY, %	24.36 ± 11.50	33.11 ± 7.40	^***^
UA, mmol/L	298.02 ± 80.89	325.73 ± 75.63	ns
TBIL, mmol/L	10.56 ± 4.81	12.50 ± 2.91	ns
AST, U/L	25.45 ± 13.77	22.63 ± 5.68	ns
ALT, U/L	25.61 ± 18.41	28.93 ± 15.44	ns
ALB, g/L	37.61 ± 4.34	38.83 ± 4.20	ns
FBG, mmol/L	5.62 ± 1.26	5.43 ± 0.38	ns
Cr, μmol/L	65.41 ± 19.66	71.93 ± 5.45	ns
CEA, ng/mL	64.79 ± 237.21	2.21 ± 0.94	ns

*WBC* White blood cell, *RBC* Red blood cell, *Hb* Hemoglobin, *NE* Neutrophils, *LY* Lymphocytes, *UA* Uric Acid, *TBIL* Total bilirubin, *AST* Aspartate aminotransferase, *ALT* Alanine aminotransferase, *ALB* Albumin, *FBG* Fasting Blood Glucose, *Cr* creatinine, *CEA* Carcinoembryonic antigen. *P* value: ns, no significance; **, *P* < 0.01; ***, *P* < 0.001

### Characteristics of exosomes derived from urine

**Fig. 2:**
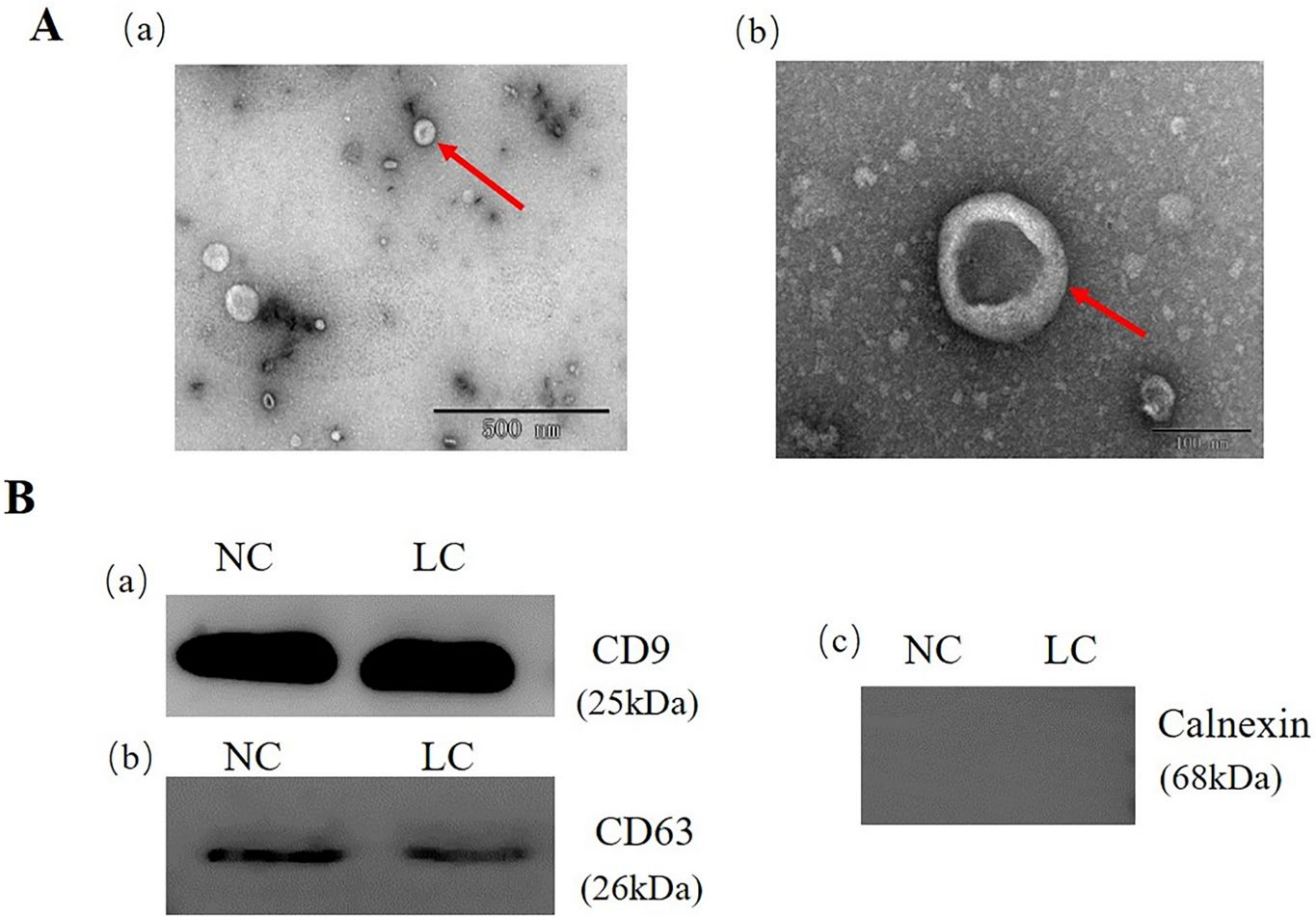
Urine exosome identification. **A** Representative TEM images of urine exosomes, scale bar; a = 500 nm, b = 100 nm. **B** Western blotting images of urine exosome marker proteins CD9 (a), CD63 (b) and Calnexin (c)

Exosomes were isolated from the urine of lung cancer patients and normal individuals, and the morphology of exosomes was detected using transmission electron microscopy (TEM), as shown in Fig. 2A. The vesicle like structure of the exosome was clearly shown in the figure. Western blotting was further used to detect the exosome markers: transmembrane proteins CD9 and CD63 (Fig. 2B). As shown, CD9 and CD63 were expressed by exosomes isolated from the urine of samples. All samples were negative for Calnexin, indicating fewer contaminants in exosomes. In accordance with the International Society for Extracellular Vesicles (ISEV) guidelines on exosome characterization, our research results proved the existence of exosomes.

### Patients with lung cancer have a reduced proportion of lymphocytes in their blood

**Fig. 3:**
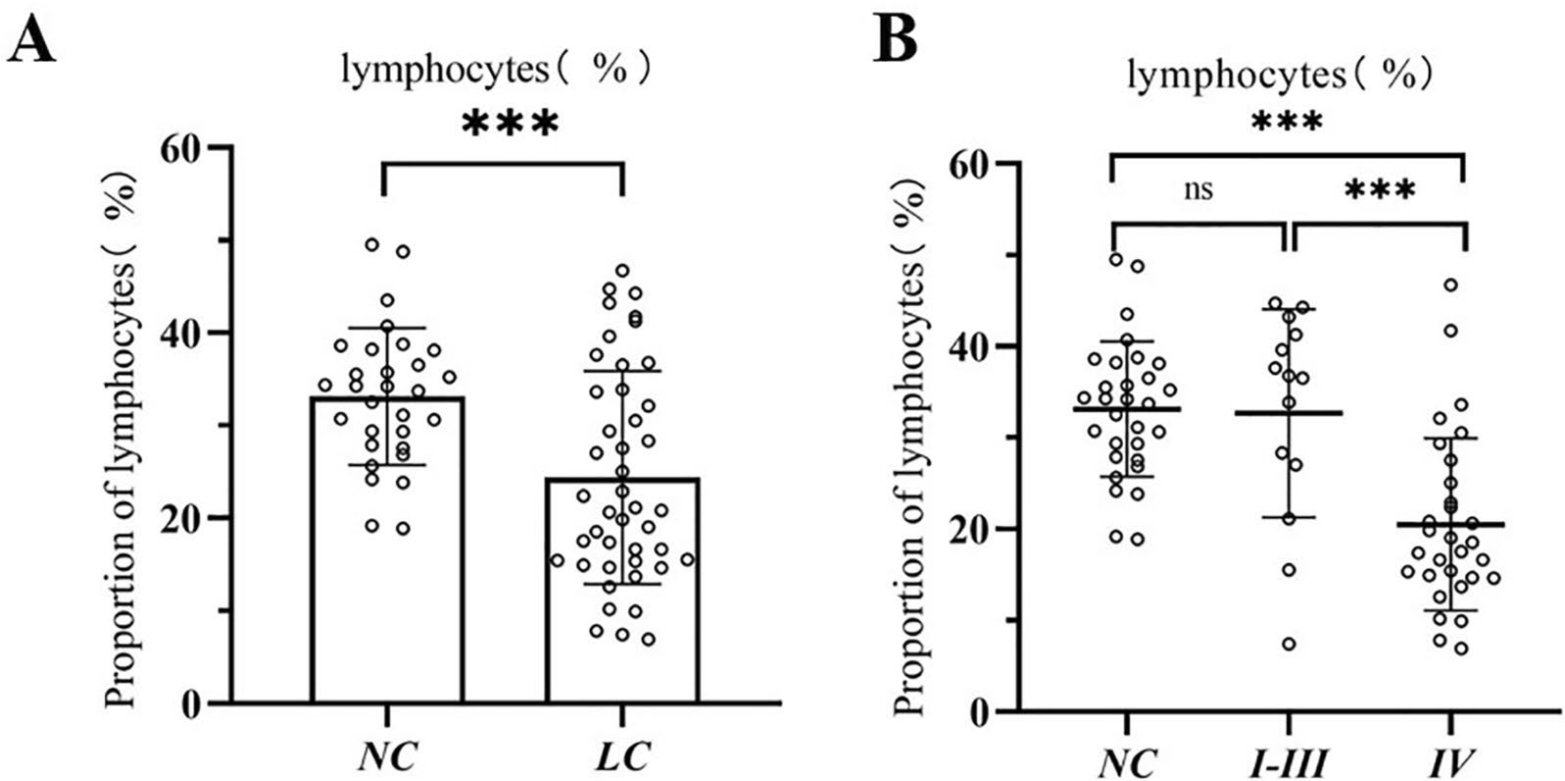
Analysis of the proportion of lymphocytes in the blood of subjects. **A** The proportion of lymphocytes in the blood of the normal control group (NC) and the lung cancer patient group (LC). **B** The percentage of lymphocytes in the blood of the normal control group (NC), the lung cancer patients with stages I-III and IV lung cancer. Symbols represent individual subjects, each measured once in independent experiments, * * *, *P* < 0.001

According to the TNM stage of lung cancer [19], we divided the samples of lung cancer group into stages I-III and IV. As shown in Fig. 3, the proportion of lymphocytes in the blood of lung cancer patients was further analyzed. The proportion of lymphocytes in the blood was decreased in lung cancer patients compared to normal controls (*P* < 0.05); the proportion of lymphocytes decreased with increasing cancer stage, and was significantly lower in patients with stage IV lung cancer compared to normal controls (*P* < 0.05).

### Mass spectrometric expression and functional analysis of lymphocyte related proteins

**Fig. 4:**
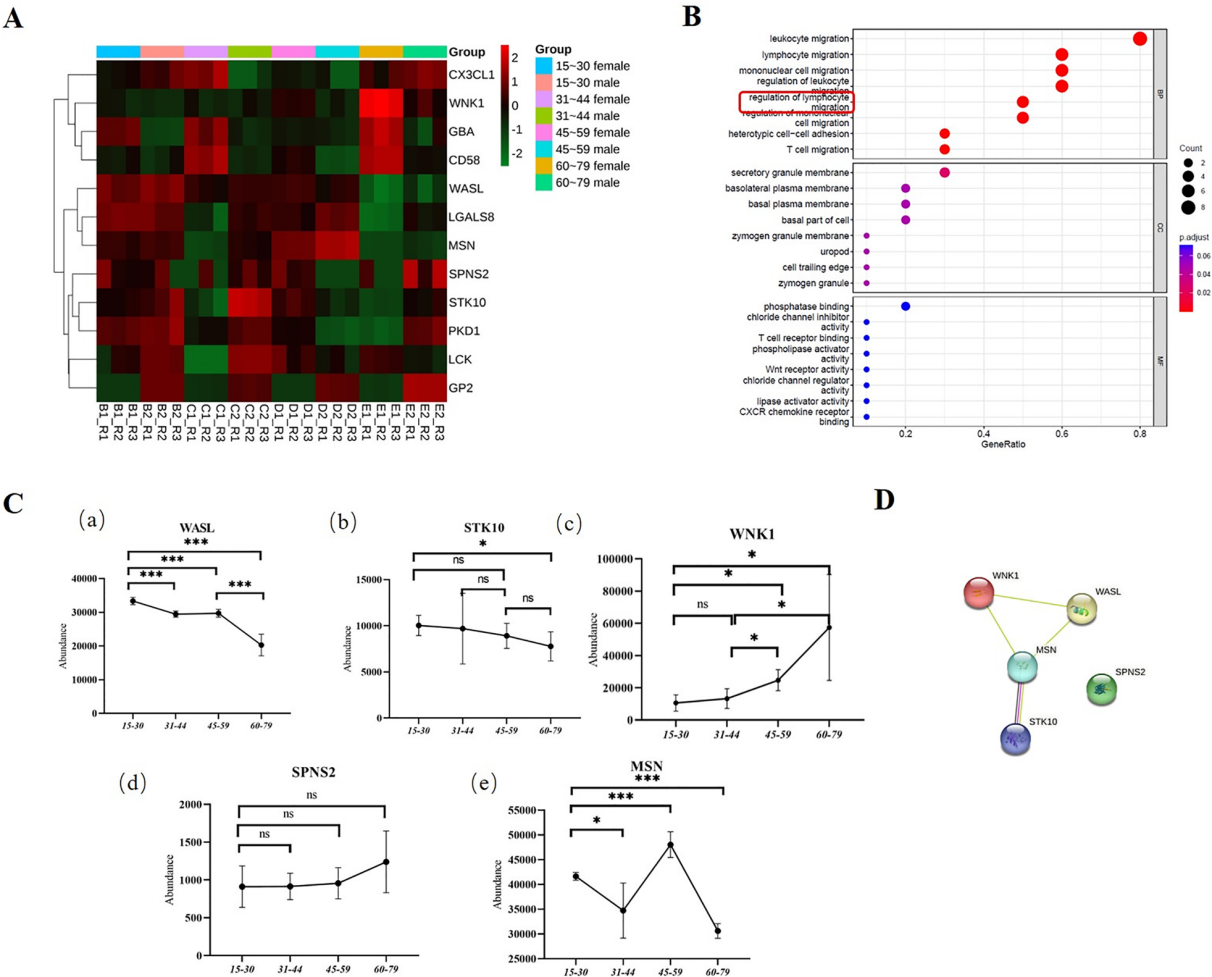
Mass spectrometric expression and functional analysis of lymphocyte related proteins. **A** Heatmap analysis of lymphocyte related proteins in urine exosomes of normal individuals aged 15–79 years. **B** GO enrichment analysis of 12 lymphocyte related proteins. Ordinates indicate go functional categories: biological process (BP), cellular component (CC) and molecular function (MF). **C** Trends in protein levels of urine exosome WASL (a), STK10 (b), WNK1 (c), SPNS2 (d), and MSN (e) in normal individuals aged 15–79 years. **D** PPI network analysis of 5 proteins related to lymphocyte migration regulation. More lines represent stronger correlations

We analyzed MS data from normal individuals and screened 12 proteins associated with lymphocytes, and specific protein information is shown in Supplementary Table 2. A heatmap of the hierarchical clustering of these proteins is shown in Fig. 4A. GO analysis was performed on these 12 proteins, and the results are shown in Fig. 4B. Biological process (BP) showed that these proteins are associated with lymphocyte migration, as well as regulation of lymphocyte migration. Among them, there are a total of 5 proteins related to the regulation of lymphocyte migration. The expression of these proteins in healthy people aged 15 to 79 years was explored, and the results are shown in Fig. 4C. In addition to MSN protein, WASL and STK10 proteins showed a gradual decrease in expression; WNK1 and SPNS2 proteins showed a gradual increase in expression with age. At the same time, the STRING database was used to predict the interactions of 5 different proteins, as shown in Fig. 4D. In addition to the SPNS2 protein, there was a correlation between WASL, STK10, WNK1 and MSN. In summary, we screened out 3 lymphocyte migration regulation related proteins for follow-up experiments, namely WASL, STK10 and WNK1.

### Differential expression of lymphocyte migration regulation related proteins in the urine exosome of lung cancer patients

**Fig. 5:**
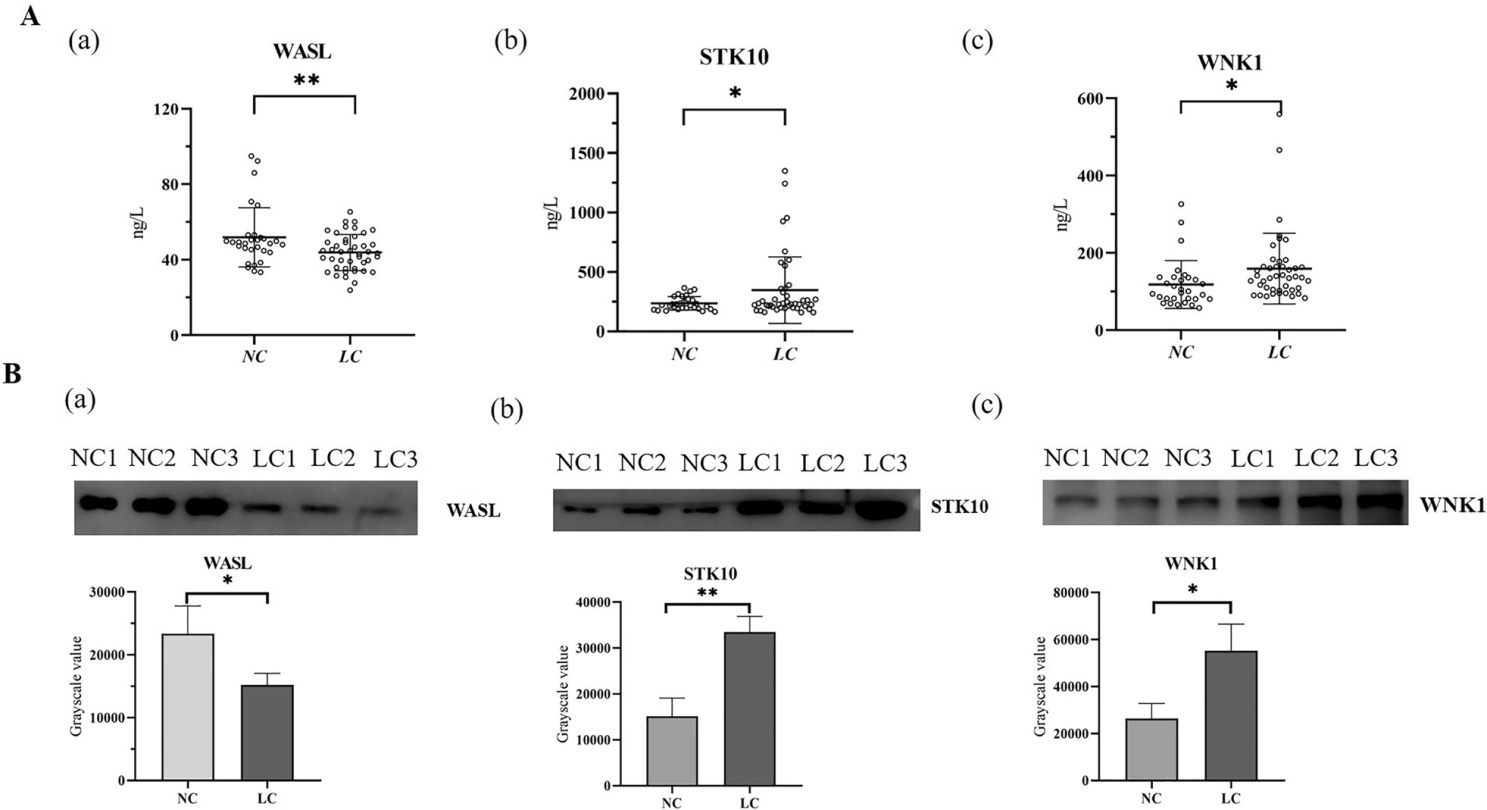
Expression of WASL, STK10 and WNK1 in urine exosomes of patients with lung cancer. **A** ELISA results of changes in WASL (a), STK10 (b) and WNK1 (c) protein concentrations in urine exosomes from normal controls (NC) and lung cancer patients (LC). Symbols represent individual subjects, each measured once in independent experiments, *, *P* < 0.05; **, *P* < 0.01; ns, not significant. **B** Images of western blotting and grayscale values for WASL (a), STK10 (b) and WNK1 (c) proteins in urine exosomes. The protein of WASL, STK10, and WNK1 were observed in the NC and LC groups

We then investigated the expression of WASL, STK10 and WNK1 in lung cancer patients. The first was the ELISA experiment, which consisted of lung cancer patients (LC, *n* = 44) and normal controls (NC, *n* = 30). As shown in Fig. 5A, the expression of STK10 and WNK1 was increased and the expression of WASL was decreased in the urine exosome of lung cancer patients compared with normal controls, with statistically significant differences (*P* < 0.05). Western blotting experiments further validated this change (Fig. 5B). Representative Western blotting bands of the three proteins are shown at the top and their grayscale value bars are shown at the bottom.

### Urine exosome regulation of lymphocyte migration related protein is valuable for the auxiliary diagnosis of lung cancer

**Fig. 6:**
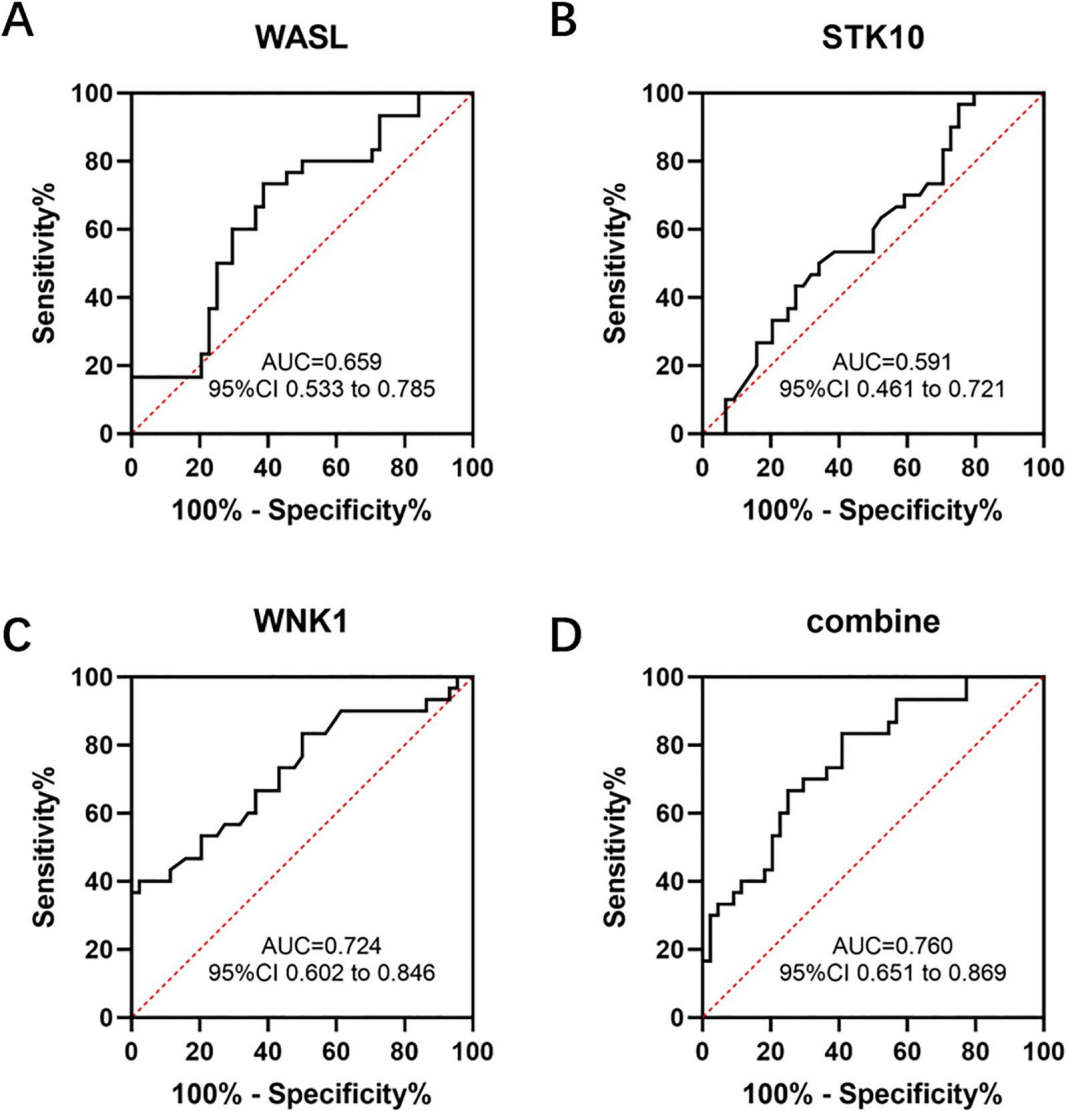
ROC curve analysis of urine exosome WASL, STK10 and WNK1 proteins. The target protein concentration was determined according to the ELISA method, and the ROC curve was plotted. ROC curves for WASL (**A**), STK10 (**B**), WNK1 (**C**), and the 3 proteins combined (**D**). AUC, area under the curve; CI, confidence interval

Based on the ELISA data, ROC curves were established to analyze the auxiliary diagnostic values of WASL, STK10 and WNK1 in the urine exosome, as shown in Fig. 6. The area under the curve of urine exosome WASL was 0.659 (95% CI, 0.533–0.785); the area under the curve for urine exosome STK10 was 0.591 (95% CI, 0.461–0.721); the area under the curve for urine exosome WNK1 was 0.724 (95% CI, 0.602–0.846). When 3 urine exosome proteins were combined, the AUC reached 0.760 (95% CI, 0.651 to 0.869), which was greater than the value of either alone.

## Discussion

Lymphocyte migration is a complex process in which multiple molecules are involved and regulated by multiple factors. A systematic and definitive understanding of this process is currently lacking. Lymphocyte migration is a research hotspot for disease treatment in the clinic. It is important to explore the expression changes of lymphocyte migration related proteins in lung cancer patients for the diagnosis of lung cancer and the selection of therapeutic targets.

Through the present study, we identified a total of 12 lymphocyte related proteins in the urine exosome, of which 5 proteins related to lymphocyte migration regulation were screened. ELISA and western blotting were used to investigate the expression changes of WASL, STK10 and WNK1 in the urine exosome of lung cancer patients. Lymphocyte migration regulation related proteins may serve as potential biomarkers for lung cancer diagnosis and have clinical applications.

WASL (WAS-like), also known as N-WASP, is an important member of the Wiskott-Aldrich family of comprehensive signature proteins [20]. WASL is directly involved in the dynamic regulation of actin filamentous branching in the cytoskeleton, closely related to the negative regulation of lymphocyte migration, and involved in the development and progression of a variety of cancers [21–23]. Differences in tumor type may contribute to differences in WASL gene function. Martin et al. identified WASL as a potential tumor suppressor gene in breast cancer [24]; WASL is lowly expressed in intestinal cancer tissues and cells and is associated with poor prognosis of intestinal cancer [25]. STK10 belongs to a family of serine/threonine protein kinases [26]. It is widely involved in the cell cycle, cell migration, and is closely related to the human immune system as well as cancer development and progression. For example, in the prostate, STK10 can inhibit prostate cancer cell proliferation and promote prostate cancer cell migration and apoptosis through p38 MAPK signaling [27]. Zhang et al. showed that loss of STK10 promotes cell adhesion, migration and invasion in cervical cancer [28]. WNK1, one of the members of the WNK kinase family, is a serine/threonine protein kinase that can be activated by phosphorylation [29]. WNK1 is aberrantly expressed in non-small cell lung cancer [30], gliomas, renal tumors [30], breast cancer, and hematological tumors. Studies have shown that elevated WNK1 expression may promote renal carcinogenesis and progression. And WNK1 is involved in bone tumor pain formation [31], providing a corresponding target for the treatment of these diseases. In this study, we identified decreased expression of WASL and elevated expression of STK10 and WNK1 in the urine exosome of lung cancer patients by detection. We speculate that this change may be related to regulation of lymphocyte migration, as it has been found that WASL is involved in the negative regulation of lymphocyte migration, whereas STK10 and WNK1 are primarily involved in positive regulation of lymphocyte migration. Low expression of WASL and high expression of STK10 and WNK1 represent increased lymphocyte migration, which may be closely related to lung cancer development and progression.

Lymphocyte migration and tumor cell metastasis share many similarities, such as the fact that both can undergo cell migration through a reversible adhesive contact process and can also exude into the surrounding tissue. Thus, it has been suggested that the tumor metastasis mimicry hypothesis, by which tumor cells adopt the behavior of mimicry, mimics many functions of lymphocytes. This completes the immune escape, leading to tumor initiation and progression. In this study, WASL, STK10 and WNK1 are involved in the regulation of lymphocyte migration. Whether the increased migration of lymphocytes in lung cancer patients is related to tumor cell migration is still unknown and requires further experimental demonstration.

Urine is an ultrafiltration of plasma, a metabolic product of the systemic organs [13]. Changes in urine exosomes represent changes in the expression of systemic proteins. We further investigated the protein concentrations of WASL, STK10 and WNK1 in urine exosomes from normal individuals at different ages (age range 15–79). Studies have shown that most of the age of onset of lung cancer is over the age of 40, and the peak incidence is mainly concentrated in the age range 60–79, which is similar to the changes of WASL and WNK1 proteins in our experimental results. We found a gradual decrease in WASL protein expression and a gradual increase in WNK1 protein expression with increasing age, and people aged 60–79 years had the lowest WASL protein and the highest WNK1 protein expression. This is consistent with the low expression of WASL and high expression of WNK1 protein in lung cancer patients, which may explain why the age of 60–79 years is the peak of lung cancer incidence.

This study is the first to report the expression of WASL, STK10 and WNK1 in the urine exosome of lung cancer patients, which may serve as potential biomarkers for lung cancer diagnosis. But at present, the protein concentration changes of WASL, STK10 and WNK1 in the urine exosome of lung cancer patients are only preliminarily explored, and their relevance to the process of cancer cell metastasis is yet to be explored, requiring more in-depth exploration.

There are some limitations to this study. The biomarkers identified in this study need to be further evaluated and validated in multiple centers and with many samples before they can be applied in clinical practice. In the future, the urine exosome proteins in this study will be applied to clinical monitoring of lung cancer to benefit a larger population.

## Conclusion

In conclusion, in the present study, we have analyzed and validated the trends in the expression of lymphocyte migration regulation related proteins from urine exosomes of lung cancer patients and normal individuals of different ages. Changes in expression of WASL, STK10 and WNK1 may serve as potential biomarkers for lung cancer diagnosis and are of great importance for the selection of therapeutic targets in lung cancer.

### Supplementary Information

**Additional file 1: Table S1.** Clinical characteristics of healthy people of different ages.

** Additional file 2: Table S2.** Specific information of 12 lymphocyte-associated proteins in urine exosomes.

**Additional file 3.** 

**Additional file 4.** 

## Data Availability

The datasets used and analysed during the current study are available from the corresponding author on reasonable request.
