# Supplementary material for: Lymphocyte migration regulation related proteins in urine exosomes may serve as a potential biomarker for lung cancer diagnosis
Source: BMC Cancer. 2023 Nov 18;23:1125. doi: 10.1186/s12885-023-11567-x (PMC10656923; doi:10.1186/s12885-023-11567-x)
Supplement: Supplementary file 1 — Additional file 1: Table S1. Clinical characteristics of healthy people of different ages. [file 12885_2023_11567_MOESM1_ESM.docx]

**Supplementary Table 1. Clinical characteristics of healthy people of different ages**

| Characteristics | 15~30 | | 31~44 | | 45~59 | | 60~79 | |
| --- | --- | --- | --- | --- | --- | --- | --- | --- |
|  | Male | Female | Male | Female | Male | Female | Male | Female |
| Age, years | 22.80±3.99 | 22.80±4.28 | 39.33±2.82 | 38.87±3.50 | 52.27±3.88 | 51.73±3.56 | 66.07±4.85 | 66.60±5.28 |
| WBC×10^9^/L | 5.75±1.07 | 5.92±1.23 | 6.06±0.92 | 6.17±1.24 | 5.47±1.20 | 4.70±1.05 | 6.00±1.24 | 5.70±1.01 |
| RBC×10^12^/L | 4.69±0.45 | 4.44±0.28 | 5.04±0.20 | 4.88±0.33 | 5.12±0.27 | 4.86±0.41 | 4.30±0.63 | 4.26±0.39 |
| Hb, g/L | 139.7±8.96 | 134.9±5.41 | 148.5±8.62 | 143.1±6.53 | 151.9±6.10 | 145.8±10.22 | 127.7±17.91 | 123.0±12.82 |
| Cr, μmol/L | 73.1±10.79 | 65.4±10.01 | 78.07±5.85 | 73.13±5.03 | 74.60±5.72 | 71.13±7.10 | 65.13±9.20 | 57.27±13.23 |
| eGFR,mL/min/1.73m2 | 121.8±19.68 | 117.3±13.25 | 108.8±8.13 | 109.6±8.10 | 96.00±5.82 | 99.53±5.15 | 93.67±29.20 | 88.13±17.88 |
| FBG, mmol/L | 5.09±0.39 | 4.93±0.49 | 5.42±0.62 | 5.32±0.36 | 5.36±0.48 | 5.15±0.35 | 5.51±0.61 | 5.90±0.81 |
| AST, U/L | 18.80±4.51 | 16.27±3.75 | 19.07±2.43 | 17.47±3.14 | 21.40±3.72 | 19.67±4.17 | 26.87±9.44 | 22.40±6.76 |
| ALT, U/L | 21.67±6.77 | 17.33±6.25 | 19.53±5.40 | 16.00±3.95 | 28.40±9.36 | 21.53±9.91 | 22.87±10.24 | 20.67±7.82 |
| Urine protein; (negative, %) | 15（100） | 15（100） | 15（100） | 15（100） | 15（100） | 15（100） | 15（100） | 15（100） |

**Note:** WBC: White blood cell; RBC: Red blood cell; Hb: Hemoglobin; Cr: Creatinine; eGFR: Estimated glomerular filtration rate; FBG: Fasting Blood Glucose; AST: Aspartate aminotransferase; ALT: Alanine aminotransferase.
