# Supplementary material for: Lymphocyte migration regulation related proteins in urine exosomes may serve as a potential biomarker for lung cancer diagnosis
Source: BMC Cancer. 2023 Nov 18;23:1125. doi: 10.1186/s12885-023-11567-x (PMC10656923; doi:10.1186/s12885-023-11567-x)
Supplement: Supplementary file 2 — Additional file 2: Table S2. Specific information of 12 lymphocyte-associated proteins in urine exosomes. [file 12885_2023_11567_MOESM2_ESM.docx]

**Supplementary Table 2. Specific information of 12 lymphocyte-associated proteins in urine exosomes.**

| **UniProt ID** | **Protein Name** | **Gene Name** |
| --- | --- | --- |
| P78423 | Fractalkine | CX3CL1 |
| Q9H4A3 | Serine/threonine-protein kinase WNK1 | WNK1 |
| P04062 | Lysosomal acid glucosylceramidase | GBA |
| P19256 | Lymphocyte function-associated antigen 3 | CD58 |
| O00401 | Neural Wiskott-Aldrich syndrome protein | WASL |
| O00214 | Galectin-8 | LGALS8 |
| P26038 | Moesin | MSN |
| Q8IVW8 | Protein spinster homolog 2 | SPNS2 |
| O94804 | Serine/threonine-protein kinase 10 | STK10 |
| P98161 | Polycystin-1 | PKD1 |
| P06239 | Tyrosine-protein kinase Lck | LCK |
| P55259 | Pancreatic secretory granule membrane major glycoprotein GP2 | GP2 |
