# Supplementary figures and images for: Lymphocyte migration regulation related proteins in urine exosomes may serve as a potential biomarker for lung cancer diagnosis
Source: BMC Cancer. 2023 Nov 18;23:1125. doi: 10.1186/s12885-023-11567-x (PMC10656923; doi:10.1186/s12885-023-11567-x)

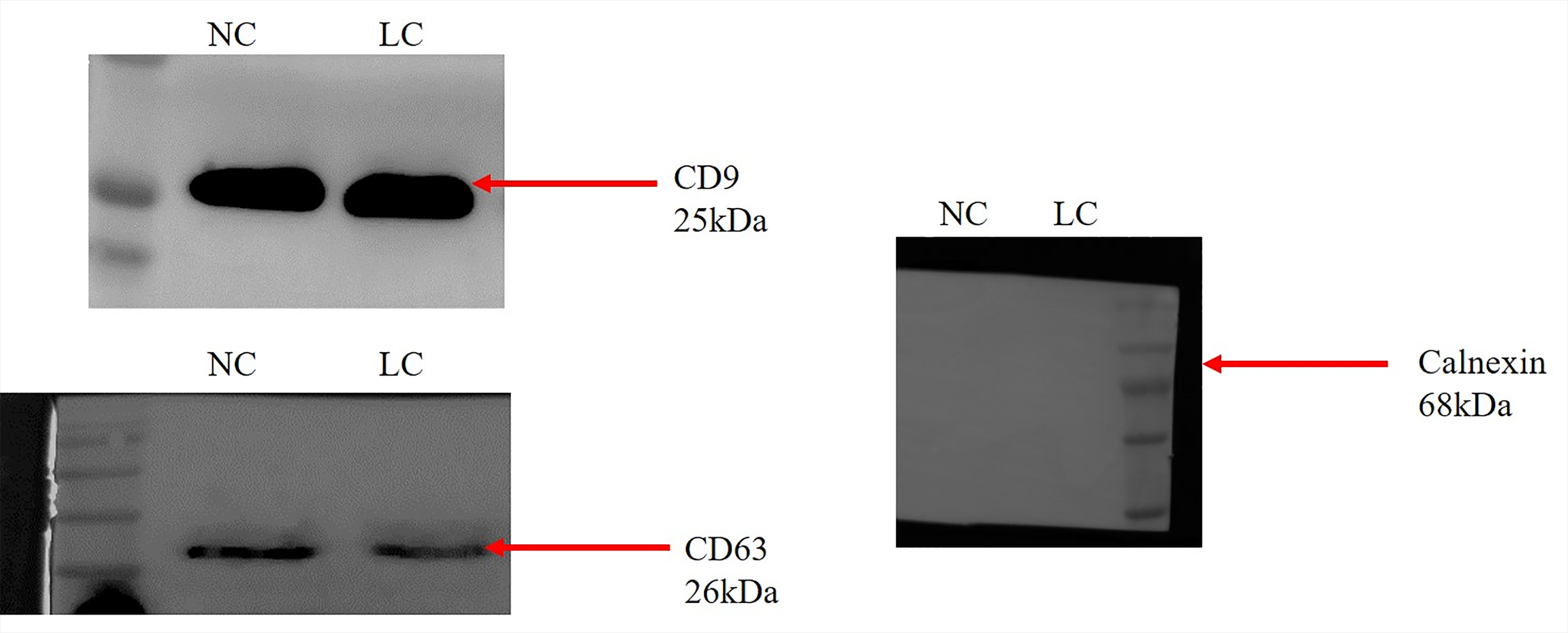

Supplement: Supplementary file 3 — Additional file 3. [file 12885_2023_11567_MOESM3_ESM.tif]

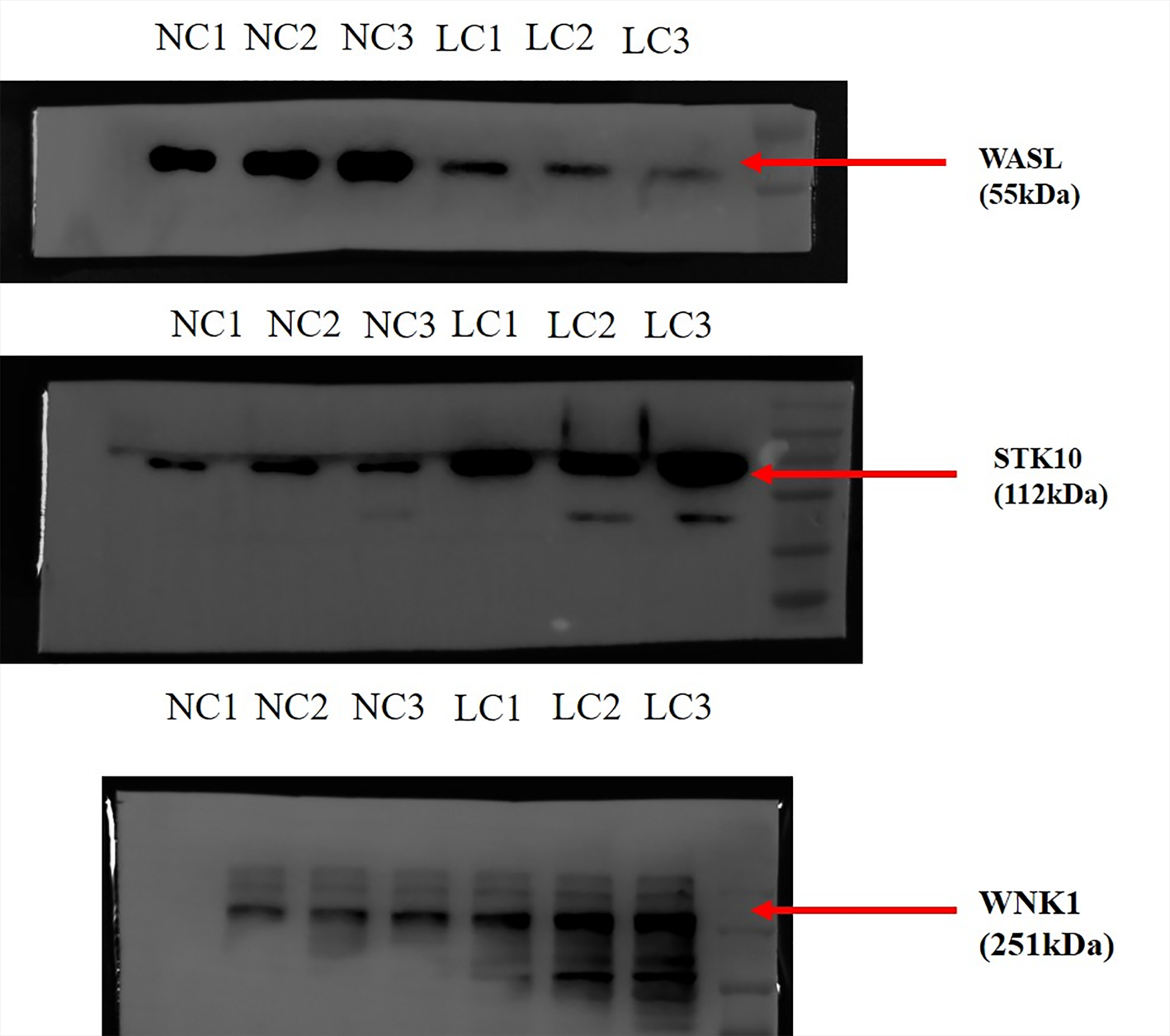

Supplement: Supplementary file 4 — Additional file 4. [file 12885_2023_11567_MOESM4_ESM.tif]
